# Increased phosphorylated tau (pTau-181) is associated with neurological post-acute sequelae of coronavirus disease in essential workers: a prospective cohort study before and after COVID-19 onset

**DOI:** 10.1016/j.ebiom.2025.106106

**Published:** 2026-01-05

**Authors:** Xiaohua Yang, Ashley Fontana, Sean A.P. Clouston, Benjamin J. Luft

**Affiliations:** a World Trade Center Health Program, Department of Medicine, Renaissance School of Medicine, Stony Brook University, NY, USA; b Program in Public Health, Renaissance School of Medicine, Stony Brook University, NY, USA

**Keywords:** Phosphorylated Tau-181, Neurological biomarkers, Post-acute sequelae of COVID-19, Coronavirus disease

## Abstract

**Background:**

The COVID-19 pandemic led to a spectrum of post-acute sequelae including several neurological complications including cognitive dysfunction labelled Neurological PASC (N-PASC). We hypothesised that N-PASC was associated with changes in neurological biomarkers after COVID-19.

**Methods:**

N-PASC was established when individuals reported accepted neurological symptoms persisting for ≥3 months arising alongside validated COVID-19. Plasma samples were retrieved from before and after COVID-19 onset among all (n = 227) essential workers who developed COVID-19 with N-PASC and demographically matched with data from 227 controls who either developed COVID-19 without N-PASC (n = 124) or did not develop COVID-19 before follow-up (n = 103). We used single molecular analysis measured pTau-181, GFAP, NfL, Aβ40/42, and total Aβ burden (IAB). Risk factors for N-PASC were examined prior to COVID-19 infection. Multivariable adjusted generalised linear longitudinal modelling with random intercepts was used to examine changes in biomarkers after COVID-19 onset.

**Findings:**

N-PASC was only associated with higher IAB before COVID-19 onset (area under the receiver-operating curve = 0.77). Longitudinal analyses revealed plasma pTau-181 levels increased by 59.3% (95% C.I. = [45.2, 73.4] P = 0.006) following COVID-19 onset in participants who developed N-PASC that were worst among participants reporting central nervous symptoms persisting ≥1.5 years. Post-COVID-19 decreased GFAP and NfL were associated with peripheral symptoms of N-PASC, but not with increased pTau-181. Having ≥20% increases in pTau-181 were associated with increased Aβ40/42 levels at follow-up, and with central neurological symptoms including lingering brain fog and loss of taste/smell.

**Interpretation:**

N-PASC with symptoms consistent with central damage were associated with increased pTau-181 levels. Increases in pTau-181 were associated with increased risk of changes to amyloid biomarkers consistent with Alzheimer's disease in participants with N-PASC and could therefore inform N-PASC prognostication.

**Funding:**

This study was supported in part by funding from the 10.13039/100000030Centers for Disease Control and Prevention (CDC/NIOSH CDC-75D30122c15522) and the 10.13039/100000002National Institutes of Health (NIH/NIA AG049953).

**Research in context**

**Evidence before this study**

A systematic literature review suggested that individuals reporting neurological post-acute sequelae (PASC) after coronavirus disease (COVID-19) have evidence of neuroinflammation and cognitive decline, while autopsy studies report evidence of vascular disease and cerebral tauopathy absent amyloidosis in non-survivors.

**Added value of this study**

This study examined biomarkers collected both prior to and following COVID-19 pandemic in a sample of essential workers at midlife to find that individuals with N-PASC had evidence of a 59.3% increase in pTau-181 levels from pre-COVID-19 levels that was not evident in other essential workers and appears to be worst among those whose N-PASC had persisted for more than 1.5 years.

**Implications of all the available evidence**

These studies imply that symptoms of N-PASC that persist for more than 1.5 years are at increased risk of developing higher than normal levels of circulating levels of pTau-181 that might portend worsened cognitive functioning as individuals age.

## Introduction

The COVID-19 pandemic affected more than 775 million individuals worldwide.^1^ Beyond the acute respiratory disease characterising primary infection with the SARS-CoV-2 virus, accumulating evidence suggests that COVID-19 might affect the central and peripheral nervous system (CNS/PNS) early in the infection course.^2^^,^^3^ Consequently, headache, encephalopathy, insomnia, stroke, and seizures have been observed in as many as 84% of patients.^4^^,^^5^

After acute symptoms resolve, some individuals continue to experience persistent post-acute sequelae of COVID-19 (PASC).^6^ N-PASC symptoms include neurocognitive changes such as brain fog, forgetfulness, or diminished executive functioning in and can persist ≥3 months.^7^ Risk factors for N-PASC include COVID-19 severity, and pre-existing medical comorbidities including diabetes, chronic obstructive pulmonary disease, and obesity,^8^ and SARS-CoV-2 re-infection.^9^ N-PASC is relatively common in individuals who developed COVID-19 before vaccination: for example, in a prospective study examining >3000 acute COVID-19 in essential workers reported that 56% exhibited neurological symptoms, and 22% developed N-PASC.^10^

Seeking an explanation for persistent neurological N-PASC (hereafter, N-PASC), researchers used neuroimaging technologies to identify diffuse neuroinflammation of the cerebral parenchyma.^11^^,^^12^ Such inflammation, when sustained, may cause persistent effects including cortical atrophy,^13^ cerebral disconnection,^14^ and blood–brain barrier disruption.^15^ A review of autopsy studies reported evidence of glial activation in patients who died from COVID-19, but also revealed that glial activation in these individuals was not associated with neurological symptoms.^16^ Neuroimaging studies suggest that neuroinflammation and concomitant brain ageing are associated with COVID-related cognitive decline in survivors, however.^17^ Interestingly, a small neuropathology study showed abnormal accumulation of hyperphosphorylated Tau protein, absent concomitant with changes in cerebral amyloidosis 4–13 months after recovery from acute COVID-19 in three patients who died after recovering from SARS-CoV-2 infection.^18^ Reiken and colleagues^19^ further examined brain lysates from autopsy specimens of patients with COVID-19 infections and found increased levels of phosphorylated tau *absent amyloidosis* alongside evidence of posttranslational modification of the ryanodine receptor, consistent with a leaky calcium channel.^20^

Tauopathy is difficult to measure, but is now being monitored using phosphorylated tau-181 (pTau-181) levels in serology,^21^, ^22^, ^23^ where researchers have reported excellent accuracy when detecting early-stage disease in the general population.^24^ We hypothesised that changes in pTau-181 levels would be associated with the onset of COVID-19 among participants who developed N-PASC. Consistent with the ryanodine receptor deletion hypothesis, we hypothesised that increases in pTau-181 would not be concurrent with evidence of increases in cerebral amyloidosis but may be concurrent with contemporaneous decreases in levels of circulating GFAP.

## Methods

### Setting

Essential workers who participate in a pre-existing health monitoring program were recruited into a SARS-CoV-2 infection identification protocol through a comprehensive questionnaire that was delivered regularly during the COVID-19 pandemic and at annual monitoring visits thereafter^25^ (Supplemental Figure S1 has temporal distribution). During an essential worker monitoring program that predated the COVID-19 pandemic, participants completed a biobanking protocol including storage of plasma during annual visits occurring from 1/2019 to 5/2024. In the present study, we selected participants whose plasma samples with one observation falling before, and one after, the COVID-19 pandemic.

We over-selected individuals with N-PASC. There was evidence of sex-differences in potential controls when comparing with versus without N-PASC, so we used propensity score matching to generate a 1:1 sample matched on demographics and medical comorbidities to a group of controls (n = 227) who either reported never having COVID-19 or who developed COVID-19 but did not report any symptoms consistent with N-PASC.

### Measures

#### N-PASC diagnosis

We followed CDC guidelines for diagnosing PASC by, first, identifying participants and collecting the date of COVID-19 symptom onset, type of acute symptom, and details about hospital admissions was collected. We verified the presence of SARS-CoV-2 infections either using polymerase chain reaction testing or antibody testing among those whose symptom onset predated test availability. A validation study examining symptom onset showed that this protocol had high accuracy to detect COVID-19-positive individuals.^25^ After vaccinations became available, initial vaccination date was recorded. COVID-19 severity was categorised into two groups based on clinical standards (none/asymptomatic/mild versus moderate/severe). Participants with validated COVID-19 were eligible for a diagnosis of N-PASC based on the continuation, or development, of ≥1 neurological symptom (e.g., loss of taste/smell, brain fog, dizziness, vertigo, tinnitus, headache, or balance dysregulation) that emerged <3 months after initial infection and persisted for ≥3 months. This diagnostic protocol had an accuracy of 91% to detect neuroinflammation when using a positron emission tomography protocol.^12^

### Primary outcomes

Neurological biomarkers, including pTau-181, are now well-established and correlate well with cerebral amyloid, pTau, and neurodegeneration.^26^^,^^27^ Briefly, participants’ plasma samples were banked in a −80 °C freezer within 30 min of blood collection and then analysed in a single batch using the ultra-sensitive SiMOA SR-X platform (Quanterix, Billerica, Massachusetts, USA). We measured the pTau-181 burden using an Advantage V2 Kit, and measured concentrations of NfL, GFAP, Aβ40, and Aβ42 in plasma using the neurology 4-plex multiplex array. Biomarker assessment succeeded in all cases for pTau-181, but failed in GFAP (n = 2), NfL (n = 4), and Aβ40/Aβ42 (n = 6). There were no statistically significant differences between individuals with successful versus unsuccessful analyses, and imputation resulted in no changes to results, so we used pairwise deletion and intent to include in all reporting.

All biomarker levels are reported as continuous variables in pg/mL. Each plate included two internal controls, and calibration covers for each biomarker.^28^ Quality control was performed and values falling outside assay detection limits were excluded from analysis. The most common way to use amyloid subspecies is to calculate the Aβ40/42 ratio,^29^ however since studies of neuroinflammatory conditions suggest that both amyloid types can diminish in serology we also calculated the inverse mean amyloid burden $\left(IAB=\frac{20}{A\beta 42+\frac{A\beta 40}{15.5}}\right)$.^29^ Higher values on all biomarkers are intended to indicate worse outcomes; to further improve comparability between biomarkers, we reported results at follow-up in terms of percent of baseline value.

We used established cutoffs to determine biomarker positivity. Chatterjee et al.^27^ suggest using cutoffs including pTau-181 ≥ 1.93 pg/mL, Aβ40/42 ratio ≥ 18.18, GFAP ≥ 183.63 pg/mL, and NfL ≥ 17.31 pg/mL to identify Alzheimer's Disease or a Related Dementia (ADRD). We also show results for a second cutoff for pTau-181 ≥ 1.93 pg/mL was validated that may be more useful in less severe conditions.^30^ There is no established cutoff for IAB, so no cutoff was reported. Since change in neuropathology in N-PASC might differ in type or severity from, we also provided results using a conservative quantitative cutoff showing the degree of increase/decrease of ≥20%, relative to baseline levels.

*Demographics* were measured before the COVID-19 pandemic and included sex (male versus female), age (in years) at time of the blood retrieval, educational attainment (high school diploma or less, some college, or university degree). Medical factors included a history of pre-COVID-19 diagnosis of diabetes, hypertension, heart disease, and the presence of obesity (>30 k/m^2^) or morbid obesity (>40 k/m^2^) as calculated using researcher-measured height and weight. Biomarker levels can be biased by blood volume,^31^ so we measured blood volume using an established protocol.^32^

*Apolipoprotein-ε4* (APOE4) allele possession is frequently used to predict the risk of Alzheimer's disease. The APOE4 genotype was measured with the Genomics Shared Resource of Roswell Park Cancer Institute with an Infinium Global Screening Array (Illumina, San Diego, CA, USA).

### Statistics

We described the sample differences when samples were retrieved before the COVID-19 pandemic. Crude differences in pre-/post-COVID-19 changes in biomarker outcomes were therefore examined with repeated measures analysis of variance (ANOVA), and P-values are reported. Multivariable adjusted changes in biomarker outcomes were measured with multilevel generalised longitudinal models.^33^ Since outcomes were skewed away from a biological floor, we followed prior studies in relying on a longitudinal multilevel log-Gamma model.^34^ Random intercepts were used in longitudinal analyses to account for individual-level variability in biomarker expression before SARS-CoV-2 infection.^35^ Beta coefficients, standard errors, and P-values derived from t-tests are reported. Results from multiple models were reported using bar graphs. For descriptive purposes, line plots and bar graphs described the association between time since infection and the change in biomarkers after infection, relying on ANOVA to examine differences in biomarker changes before 1.5 years, and afterwards. Since we were interested in the prevalence of large changes in biomarker values, we used a quantitative cutoff (relative change ≥20% of baseline values). Logistic regression is biased in analyses where outcomes are common (>5%), so we used robust log-Poisson regression to estimate multivariable-adjusted relative risks (aRR) and accompanying 95% confidence intervals [95% C.I.].^36^ We used robust log-Poisson models to examine the risk of specific N-PASC symptoms in this cohort and examined the extent to which those symptoms were associated with biomarker changes. In all models, we used a two-tailed statistical testing (α = 0.05) and reported exact values; we adjusted P-values for the false discovery rate where necessary (FDR = 0.05). Analyses were performed in Stata 17/MP [StataCorp].

A simulation power analysis (power = 0.80, α = 0.05) suggested that a longitudinal model would require a sample size ≥205 participants with N-PASC to determine a difference of ≥0.125 SDs, and ≥430 participants (half with N-PASC) to identify differences between groups over time.

### Supplemental analyses

We examined the extent to which biomarkers differed between groups at baseline. We also examined the degree of agreement or association between individuals with biomarkers across different affected biomarkers. Finally, though not relevant to COVID-19 because we were examining biomarkers of ADRD, we also examined the extent to which APOE4 allele possession was associated with changes in biomarkers.

### Ethics

The Committee on Research Involving Human Participants reviewed and approved this study (IRB#604113); all study procedures were completed following the protocol. All participants provided informed written consent. This report follows STROBE reporting guideline for cohort studies.^37^

### Role of the funder

The funding agency played no role in analysis or writing and did not influence the decision to submit for publication. The sponsor was not involved in the study design or conduct, data collection or analysis, reporting, or in the decision to submit this manuscript for publication.

## Results

After matching and application of inclusion/exclusion criteria (Supplemental Figure S2), a total of 227 paired samples were collected from individuals with COVID-19 and were matched to 227 participants controls, 124 of whom reported a COVID-19 infection without any persistent symptoms, and 103 reported no COVID-19 infections between two biomarker measurements both occurring prior to vaccination.

Descriptive characteristics (Table 1) showed that participants with N-PASC were in their mid-fifties (56.1 ± 7.6 years) and revealed no statistically significant differences between groups. Not shown, the average N-PASC was assessed 2.7 ± 0.7 years after N-PASC symptom onset.

**Table 1:** Acute and residual symptoms of COVID-19 in participants with N-PASC as compared to participants without N-PASC.

Characteristics	N-PASC (n = 227)	Non-PASC (n = 227)	P-value
Age, years	56.05 (7.57)	55.59 (7.48)	0.499
**Sex**			
Female	19 (8.37%)	12 (5.29%)	–
Male	208 (91.63%)	215 (94.71%)	0.193
**Educational attainment**			
High school or less	49 (21.59%)	47 (20.7%)	–
Some college	106 (46.7%)	121 (53.3%)	0.660
University degree	72 (31.72%)	59 (25.99%)	0.550
**Race/Ethnicity**			
Non-Hispanic white	202 (88.99%)	196 (86.34%)	–
Hispanic	21 (9.25%)	26 (11.45%)	0.530
Other	4 (1.76%)	5 (2.2%)	0.340
**Medical comorbidities**			
Diabetes	25 (11.01%)	34 (14.98%)	0.166
Heart disease	7 (3.08%)	7 (3.08%)	1.000
Hypertension	83 (36.56%)	75 (33.04%)	0.556
Obese	111 (48.9%)	101 (44.49%)	0.186
Morbidly obese	21 (9.25%)	15 (6.61%)	0.230
†APOE4 allele possession	11 (15.71%)	28 (28.87%)	0.047
**Acute COVID-19 characteristics**			
Vaccinated before infection	18 (7.93%)	31 (13.66%)	0.050
Date of diagnosis	12/6/20	12/31/20	0.344
Date of reported symptom onset	10/28/20	11/22/20	0.263
Moderate/Severe COVID-19	132 (58.2%)	56 (38.6%)	<0.001
Vaccinated at infection	0 (0%)	27 (11.89%)	<0.001
Vaccinated at follow-up	186 (81.9%)	112 (77.2%)	0.165
Hospitalised	45 (19.8%)	13 (9.0%)	0.073
Likely Variants			
Wild (Sx before 8/24/2020)	81 (37.5%)	41 (33.1%)	–
Alpha (8/25/2020–3/14/2021)	104 (48.1%)	52 (41.9%)	0.962
Delta (3/15/2021–12/4/2021)	22 (10.2%)	19 (15.3%)	0.145
Omicron (Sx after 12/5/2021)	9 (4.2%)	12 (9.7%)	0.040
**Acute COVID-19 symptoms**			
Fever	132 (58.1%)	47 (32.2%)	<0.001
Fatigue	159 (70%)	56 (38.8%)	<0.001
Headache	128 (56.4%)	45 (31.3%)	<0.001
Cough	142 (62.6%)	42 (28.6%)	<0.001
Chills	63 (27.8%)	18 (12.3%)	<0.001
Joint/Muscle pain	135 (59.5%)	48 (33%)	<0.001
Constipation	83 (36.6%)	19 (13.2%)	<0.001
Wheeze	78 (34.4%)	15 (10.6%)	<0.001
Sore throat	75 (33%)	24 (16.3%)	<0.001
Loss of taste or smell	129 (56.8%)	31 (21.6%)	<0.001
Gastrointestinal	118 (52%)	62 (42.7%)	0.048
Shortness of breath	125 (55.1%)	25 (17.2%)	<0.001
Nausea or vomiting	19 (8.4%)	7 (4.8%)	0.131
Dizziness/Vertigo	135 (59.5%)	55 (37.9%)	<0.001
Anxiety	23 (10.1%)	0 (0%)	<0.001
Brain fog	33 (14.5%)	1 (0.9%)	<0.001
Congestion	32 (14.1%)	16 (11%)	0.321
Weight loss	17 (7.5%)	3 (2.2%)	0.009
**N-PASC symptoms**			
Brain fog	143 (63%)	0 (0.0%)	<0.001
Anosmia/Ageusia	125 (55.1%)	0 (0.0%)	<0.001
Behavioural change	43 (18.9%)	0 (0.0%)	<0.001
Gastrointestinal	30 (13.2%)	0 (0.0%)	<0.001
Vertigo	25 (11%)	0 (0.0%)	<0.001
Tinnitus	11 (4.8%)	0 (0.0%)	<0.001
Dizziness	16 (7%)	0 (0.0%)	<0.001
Loss of balance	5 (2.2%)	0 (0.0%)	0.045

**Note:** N-PASC: post-acute sequelae of COVID-19; COVID-19 coronavirus disease 2019. P-values were estimated using Carmitage's non-parametric trend test. Note that APOE4 allele possession was only available for 167 participants.

**Fig. 1:**
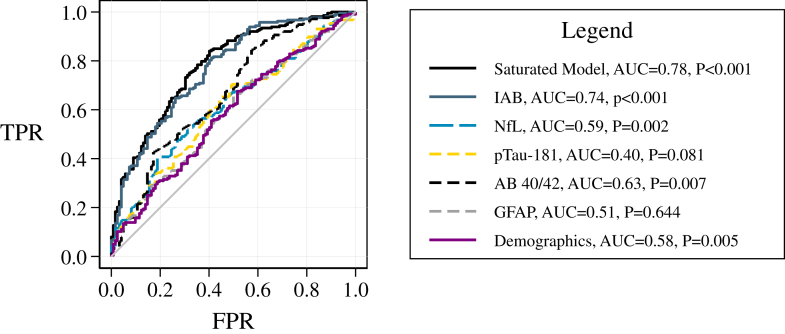
**Area under the receiver operating curve (AUC) showing the ability for pre-COVID-19 differences in each biomarker to predict the development of PASC after COVID-19 infection in the sample of individuals who developed COVID-19 between the first and second observation**.

Fig. 1 shows area under the receiver operating curve determining potential pre-COVID-19 risk factors for the development of N-PASC after COVID-19 (maximal AUC = 0.78; largest single-biomarker risk factor was IAB, AUC = 0.77). In the saturated model adjusting for demographics and biomarkers, analyses suggested that IAB (B = 4.20 [2.99–5.41] P < 0.001) and Aβ40/42 (B = −0.02 [−0.036, −0.003] P = 0.019), but not pTau-181 (P = 0.270), were elevated in participants who developed N-PASC.

**Fig. 2:**
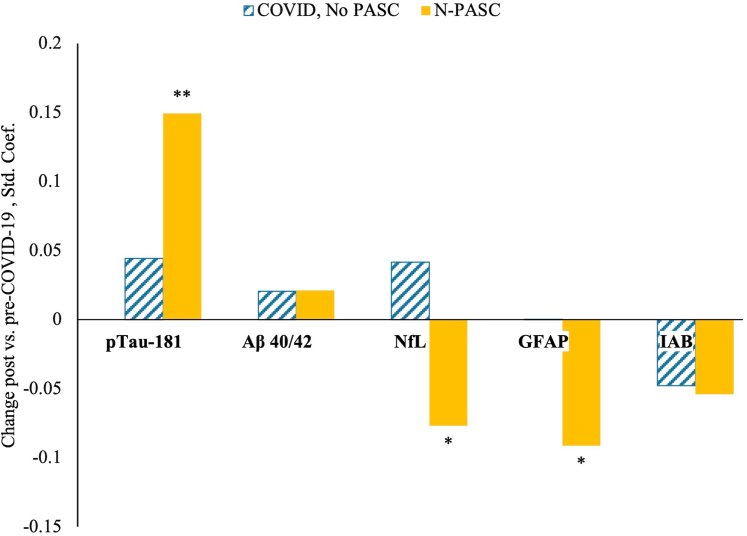
**Standardised beta coefficients establishing longitudinal rates of change after the onset of COVID-19, stratified by biomarker.** Analyses were grouped to compare differences between individuals who developed COVID-19 but did not develop N-PASC (turquoise dashed bars), and those who did develop both COVID-19 and N-PASC (gold solid bars). The reference category is individuals who were followed-up twice over the same period but who did not develop COVID-19 (set at zero). Coefficients are derived from multiple biomarker-specific ln-Gamma models and adjust for age, gender, and blood volume levels, and individual differences in pre-COVID-19 proteomic regulation propensity. ∗∗FDR-Adjusted P-value < 0.05, ∗P < 0.05.

In longitudinal analyses, pTau-181 showed a statistically significant longitudinal increase (β = 0.15, P < 0.001) in the N-PASC group when compared to controls (Fig. 2) corresponding with a 0.24 pg/mL increase over baseline or a 59.3% increase (95% C.I. = [45.2, 77.34], P < 1E-06; full results in Supplemental Table S1). When compared to never-COVID-19 controls, we also found statistically significant reductions in the GFAP (β = −0.091, P = 0.007) and NfL (β = −0.077, P = 0.022) among those with N-PASC. Among participants infected with COVID-19, we also saw an increase in IAB when compared to never-infected controls (β = 0.19, p < 0.001).

**Fig. 3:**
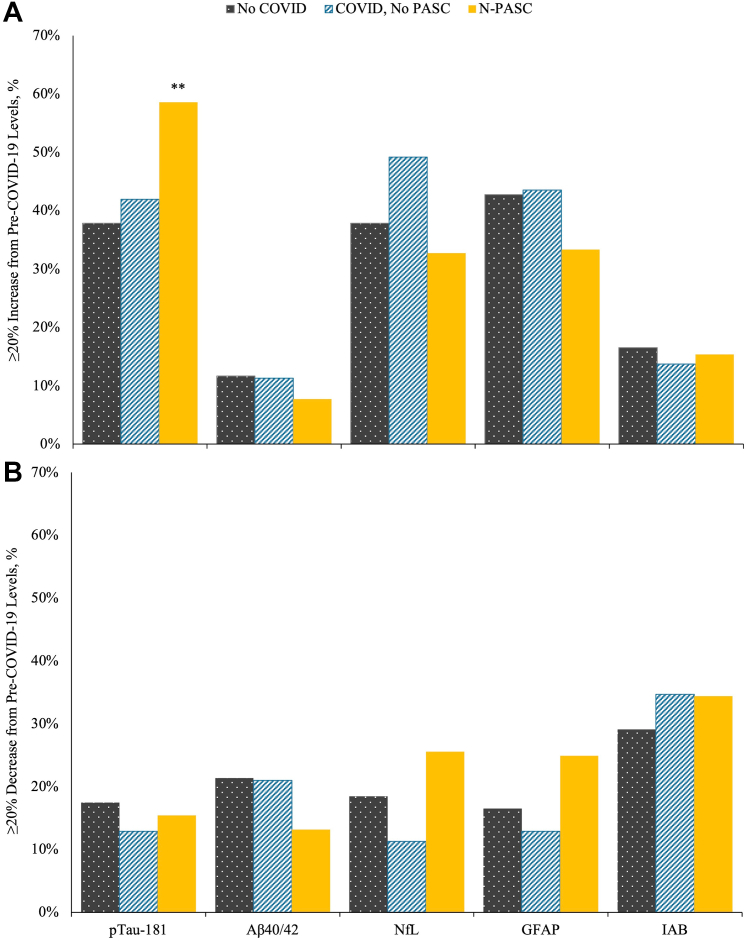
**Prevalence of increases and decreases in biomarker levels at follow-up across COVID-19 groupings.** Panel A shows ≥20% relative increases in biomarker levels from baseline. Panel B shows ≥20% relative decreases in biomarker levels from baseline, grouped by biomarker type. Estimates stratified to compare differences between individuals without COVID-19 (grey, dotted bar), those who developed COVID-19 but not N-PASC (turquoise dashed bars), and those who did develop COVID-19 and N-PASC (gold solid bars). ∗∗FDR-Adjusted P-value < 0.05, ∗P < 0.05.

Fig. 3 illustrates the number of participants whose values on biomarkers changed from baseline, stratified by COVID-19 infection and N-PASC. Overall, 58.6% of N-PASC participants exhibited increases in pTau-181 levels ≥20% relative to pre-COVID levels. Interestingly, 25.8% of participants with N-PASC experienced ≥20% relative decreases in GFAP from pre-COVID-19 levels, while 25.6% of participants experienced ≥20% relative decreases in NfL from pre-COVID-19 levels. Though not shown in Fig. 3, having a ≥20% increases in pTau-181 levels was associated with higher risk (RR = 2.16 [1.40–3.33] P = 0.001) that pTau-181 levels were deemed abnormal (pTau-181 > 1.93, prevalence = 45.1% in participants with N-PASC), and while there was a trend linking pTau-181 increases with heightened Aβ40/42 Ratio (RR = 1.83 [0.99–3.38] P = 0.054). As shown in Supplemental Table S2, when examining crossover between biomarkers we found that 75.6% of participants with N-PASC had exhibited increased pTau-181 (57.6% showed this phenotype) with/without decreased GFAP or NfL (39.3% showed one of these phenotypes). Also not shown in Fig. 3, analyses relying on biomarkers at baseline to predict change in pTau-181 levels at follow-up revealed that PASC (β = 17.26, SE = 4.73, P < 0.001) and IAB values (β = 184.29, SE = 75.22, P = 0.014) were both associated with the degree of pTau-181 change at follow-up.

**Fig. 4:**
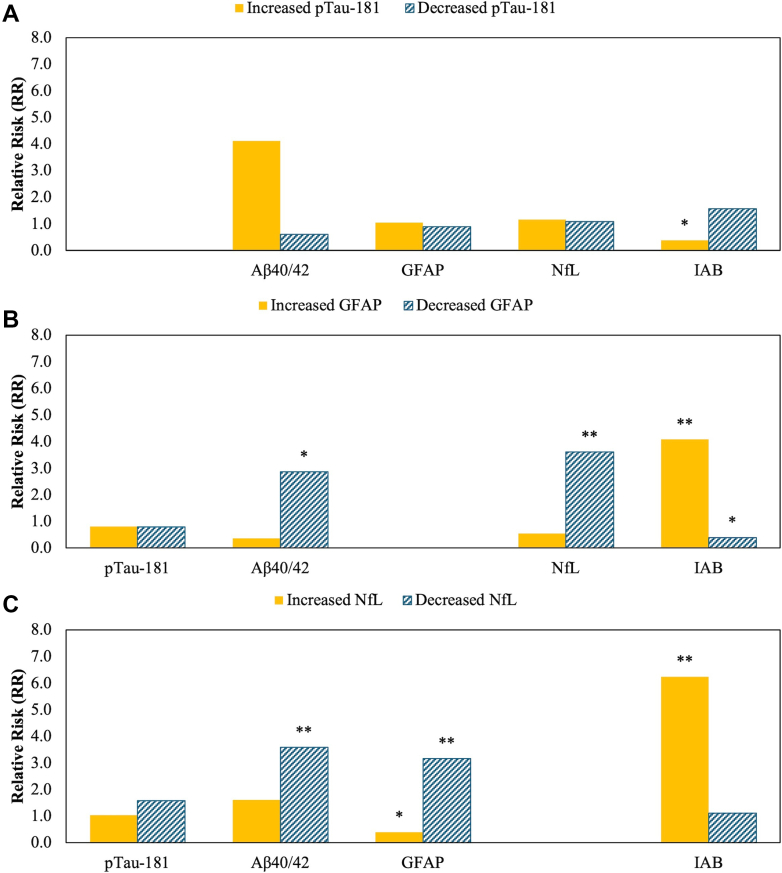
**Overlap in change in biomarkers after the onset of N-PASC.** Results are stratified by colour so that ≥20% increases in biomarkers (gold) versus ≥20% decreases in biomarkers (striped, turquoise). Note that results are missing when comparing relative risks in relation to increases/decreases in the same biomarker. Panel A shows results for N-PASC-related increases in pTau-181. Panel B shows results associated with N-PASC-related decreases in GFAP. Panel C shows associations with N-PASC-related decreases in NfL. ∗∗FDR-Adjusted P-value < 0.05, ∗P < 0.05.

Fig. 4 shows relative risks linking ≥20% increases in pTau-181 or decreases in GFAP and NfL in participants with N-PASC. These findings revealed that increases in pTau-181 was associated with a strong increase in Aβ40/42 ratios (RR = 8.16 [1.03–64.87] P = 0.048), alongside increases in the risk that IAB increased ≥20% (aRR = 1.68 [1.04–2.71] P = 0.036), alongside a concomitant decreased risk that IAB decreased by ≥ 20%. In contrast, ≥20% relative decreases in GFAP and NfL were intercorrelated (aRR = 2.85 [1.52–5.33] P = 0.001) and were associated with a very high risk that IAB increased ≥20%. Decreases in NfL were associated with ≥20% decreased Aβ40/42 ratios (Fig. 4).

Table 2 shows the degree of association between biomarker changes in N-PASC and specific symptoms. These results highlight that pTau-181 changes were mostly associated with evidence of symptoms consistent with neurological changes including muscle weakness (aRR = 1.30 [1.08–1.57] P = 0.006) alongside loss of taste/smell, anxiety/depression, and brain fog. Intriguingly, reductions in GFAP were most strongly associated with evidence of muscle weakness (aRR = 1.69 [1.17–2.46] P = 0.006) alongside fatigue, and loss of taste/smell but decreases in NfL were only associated with residual shortness of breath (aRR = 1.76 [1.07–2.90] P = 0.028).

**Table 2:** Association between specific lingering symptoms of coronavirus disease 2019 (COVID-19) and increases in pTau-181 or decreases in GFAP/NfL.

Residual symptom	≥20% increase in pTau-181		≥20% decrease in GFAP		≥20% decrease in NfL	
	aRR 95% C.I.	P	aRR 95% C.I.	P	aRR 95% C.I.	P
Any central nervous system	1.31 (1.09–1.58)	0.004	1.22 (0.78–1.91)	0.302	1.22 (1.22–1.22)	0.246
Any peripheral nervous system	1.28 (1.04–1.56)	0.018	1.22 (0.57–2.61)	0.015	1.23 (1.23–1.23)	0.064
Lost sense of taste/Smell	1.30 (1.07–1.59)	0.010	1.57 (1.05–2.35)	0.029	1.24 (0.80–1.92)	0.327
Anxiety or depression	1.26 (1.05–1.53)	0.016	1.21 (0.82–1.78)	0.343	1.13 (0.76–1.68)	0.556
Brain fog	1.22 (1.00–1.48)	0.047	1.21 (0.81–1.81)	0.352	1.31 (0.88–1.95)	0.185
Headache	1.13 (0.69–1.84)	0.630	1.51 (0.64–3.56)	0.346	1.41 (0.6–3.29)	0.427
Muscle weakness	1.30 (1.08–1.57)	0.006	1.69 (1.17–2.46)	0.006	1.38 (0.95–2.01)	0.090
Fatigue	1.20 (0.91–1.58)	0.191	1.67 (1.05–2.67)	0.031	1.42 (0.85–2.37)	0.179
Cough	1.12 (0.79–1.58)	0.536	1.28 (0.65–2.50)	0.472	1.28 (0.64–2.55)	0.481
Shortness of breath	1.19 (0.90–1.56)	0.217	1.46 (0.85–2.49)	0.167	1.76 (1.07–2.90)	0.028
Wheezing	1.27 (0.82–1.97)	0.282	0.90 (0.25–3.26)	0.874	1.34 (0.48–3.73)	0.574
Congestion	1.39 (0.97–2.00)	0.072	0.85 (0.24–3.01)	0.795	0.40 (0.06–2.64)	0.343

**Note:** Results report results after adjusting for demographics. Peripheral symptoms included numbness and tingling or pain in the extremities. Central symptoms included brain fog, loss of taste or smell, dizziness, tinnitus, loss of balance, and vertigo.

**Fig. 5:**
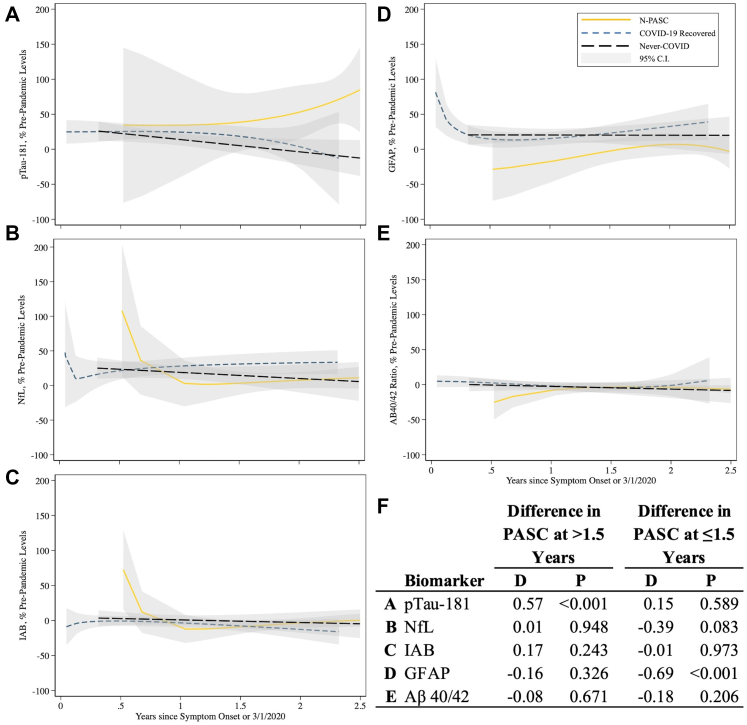
**Best fitting fractional polynomial time curves stratified by the incidence of COVID-19 and the presence of N-PASC.** Three groups include individuals who developed a validated case of acute COVID-19 and developed subsequent N-PASC (gold solid line), individuals who developed a validated case of acute COVID-19 but who did not develop any N-PASC (navy dashed line), and post-pandemic values for individuals who had not yet developed COVID-19 (charcoal long-dashed line). 95% confidence intervals are shown in transparent grey boxes. Panels A–E show the temporal trajectories of each biomarker, while Panel F shows the estimated differences before/after 1.5 years after COVID-19. Note reports the average difference in levels of change from normal among individuals whose N-PASC has lasted more, or less, than 1.5 years.

Next, we showed the mean elevation in biomarkers by time since COVID-19, stratified by infection and N-PASC status (Fig. 5). These analyses suggested that while the increase in pTau-181 levels was, on average, only 14.6% within ≤1.5 years of infection but increased 56.5% (P < 0.001) among individuals with N-PASC who were assessed >1.5 years after symptom onset. Similarly, reductions in GFAP were more pronounced among participants with N-PASC ≤1.5 years after infection (69.0% decrease, P < 0.001 versus 16.3% decrease, P = 0.33 among those within >1.5 years of infections).

*Supplemental analyses* examined whether APOE4 genotype was associated with increases in pTau-181 levels in N-PASC. In 167 participants who had agreed to genotyping, we found that APOE4 possession was not associated with change in any of the biomarkers among individuals with N-PASC (P-values > 0.10), but was generally associated with higher pTau-181 levels (P = 0.002 at baseline, P = 0.006 at follow-up in participants with N-PASC).

## Discussion

In this moderately-sized prospective study of patients who developed N-PASC we reported that, when compared to pre-COVID-19 information, levels of pTau-181 (a biomarker of neurodegenerative diseases) increased more among participants with N-PASC than among similar individuals who, at the time of data collection, had not developed COVID-19 or had developed an acute case of COVID-19 that lacked residual symptoms. Results are consistent with the view that changes in pTau-181 inconsistent with normal ageing may be common among participants with N-PASC. Indeed, more than half of participants with N-PASC experienced a ≥20% increase in absolute levels of pTau-181 relative to pre-COVID-19 levels. Among participants with N-PASC who exhibited pTau-181 increases ≥20% relative to pre-COVID levels, 45.1% expressed pTau-181 levels above an established cutoff to identify ADRD.^30^ Examining progression, increases in pTau-181 levels were higher among those with N-PASC duration periods >1.5 years since infection among those with N-PASC, consistent with a heightened potential for longitudinal progression, and increases were also associated with increased risk of abnormal AB40/42 ratios consistent with Alzheimer's disease.^27^

N-PASC was associated with changes in pTau-181 that exceeded cutoffs used in studies of ADRD. Several studies have noted evidence of change to cognition after SARS-CoV-2 infections emerging among those with N-PASC.^38^ Evidence from biomarker studies is clear in indicating that increases in the Aβ40/42 ratio alongside increases in pTau-181 are phenotypic of sporadic ADRD,^27^ and may portend a worsening prognosis in a subset of individuals. If prognosis is poorer, then researchers may expect that studies reporting COVID-19 related cognitive decline may find worsening functioning over longer follow-up periods.^38^, ^39^, ^40^ Further research is warranted to determine the cognitive implications of biomarker dysregulation in N-PASC.

The findings of persistent elevation of plasma pTau-181 support the conclusion that pTau-181 may identify a role of sustained tau pathology after infection. The fact that average elevation in pTau-181 grew over the observational period may, if replicated longitudinally, suggest a temporal lag between COVID-19 onset and increases in circulating levels of tau phosphorylation. These findings support pTau-181 as a valuable longitudinal biomarker for N-PASC and highlight the potential need for early, tau-targeted interventions to mitigate progressive cognitive decline. However, these results also require replication in neuroimaging to both clarify whether elevations for pTau-181 indicate the presence of cerebral tauopathy and, if so, what type while also determine the extent to which stability in pTau-181 indicate the absence of cerebral tauopathy N-PASC rather than biomarker insensitivity or random error over time.

Our study found that patients who develop N-PASC after COVID-19 might share certain clinicopathological features with AD. Indeed, while Tau functions adaptively to stabilise neuronal microtubules under normal physiologic conditions^23^ and can also be dysregulated and spread by reactive glia,^41^ especially in the context of chronic inflammation.^42^ However, the prognostic implications of increases in circulating pTau-181 absent concurrent amyloidosis are unknown. Usually, β-amyloid peptide plays a central role in triggering Tau phosphorylation in ADRD,^43^ a process that leads to subsequent microtubule destabilisation resulting in threads that coalesce into neuronal tangles.^44^ Intriguingly, in this study we found that evidence of increased pTau-181 was associated with increases in AB40/42 ratios consistent with a pTau-mediated ADRD. Further studies are needed to determine whether the increased levels of plasma pTau-181 correlate with evidence of cerebral Tauopathy and, if results are replicated, pTau-181 might aid in diagnosis and might serve as an important monitoring and therapeutic target.^45^

We found that individuals who developed N-PASC had higher Aβ40/42 ratios (AUC = 0.63, P = 0.007), NfL (AUC = 0.59, P = 0.002), and IAB values (AUC = 0.74, P < 0.001) before developing COVID-19. Higher values suggest that N-PASC might be more likely in those individuals who have heightened vulnerability to neurological disease. Amyloidosis often requires a secondary neuropathology to elicit the most severe symptomatology. If these findings indicate that cerebral amyloidosis is present, even in its mildest forms, then the post-COVID-19 increase in pTau-181 may correspond to the onset of pathological Alzheimer's disease.

One potentially paradoxical finding was that higher pre-COVID-19 NfL was weakly associated with higher risk of N-PASC but that individuals with N-PASC then saw decreased NfL and GFAP following COVID-19 onset appears paradoxical. We did not find that this was associated with evidence of increased pTau-181 but, instead, found that the decrease in NfL was coupled with reduced GFAP. One possibility is that reductions in GFAP and NfL indicate that the neuroimmune system is repairing itself and utilising these proteins to aid in neurogenesis, but if so the presence of persistent symptoms seems unlikely and we would expect to see glial activation in autopsy studies, a result that has not been identified.^18^^,^^19^ An alternative explanation may be that in some individuals, COVID-19-related neuroinflammation restricted glymphatic clearance causing increased aggregation of larger proteins (like NfL or GFAP: width ∼10 nm) but potentially allowing smaller proteins like pTau-181 (5 nm) to pass through unimpeded. Further studies are needed that seek to determine the impact of N-PASC on glymphatic clearance to determine if it, and not reductions in NfL and GFAP, are also associated with peripheral nervous and cerebrovascular systems.

Evidence suggests that COVID-19 involves the neuroimmune system in a heretofore unrecognised way and as the immune response systematically evolves, the infection resolves.^46^^,^^47^ However, perhaps because of the immunologically privileged nature of the central nervous system, infections may persist and give rise to indolent subacute encephalitis with concomitant neuroinflammation.^48^ Prior neuroimaging studies have demonstrated that patients with N-PASC show diffuse changes in white and grey matter connectivity, neuroinflammation, and cerebral atrophy Molecular imaging studies have revealed diffuse microglial activation.^11^^,^^12^ Yet, microglial activation is known to facilitate the spread of pre-existing cerebral tau across neurons, so perhaps activated microglia release inflammatory cytokines to trigger kinases responsible for tau phosphorylation and accelerate progression of latent neuropathology.^49^

### Limitations

This study nevertheless has several important limitations. First, this study used information on plasma distribution occurring before the COVID-19 pandemic to help develop biomarker-related results and provide evidence of mild to severe increases in pTau-181 after N-PASC development. Studies relying solely on post-COVID information might have diminished effect sizes, because they cannot ensure normal pTau-181 levels in participants before infection.

Second, this study relied on a sample of essential workers who participated in an occupational monitoring study. Since N-PASC in this population was diagnosed prospectively as individuals experienced a pandemic, and COVID-19 diagnoses were verified by medical charts, this study is likely to provide more reliable and sensitive results than other studies.

Third, because the study was limited to several types of essential workers, relatively few women were included. Despite the fact that women make up the majority of participants in studies of N-PASC, in our study male participants tend to have more severe COVID-19,^50^ but clinical studies have noted that women are about 90% more likely to report having ≥3 N-PASC symptoms and carry a higher levels of post-COVID fatigue.^51^ Since women are also known to have higher burden of cerebral tau,^52^ more work is needed that specifically focuses on sex differences in N-PASC.

Fourth, while examining several cognitively active phenotypes in these data, we did not examine cognition in this study. Future work is needed not only to examine whether concurrent changes in biomarkers mediate the established relationship between N-PASC and cognition but also whether changes in biomarkers portend a pattern of progressive decline consistent with ADRD as was implicated by concurrent elevations in pTau-181 and the Aβ40/42 ratio.

Fifth, while results for GFAP and NfL appear to go in an incorrect direction, it is worth noting that GFAP and NfL may be expressed by other tissues including in the reproductive organs (GFAP, NfL) and eyes (NfL) and so decrements in the blood may not directly reflect changes in the brain. Future research is needed that specifically examines whether changes in the blood are correlated with similar changes evident in the brains of individuals with N-PASC.

Finally, while we examined a biomarker for cerebral tauopathy, this study did not test whether tau in N-PASC was associated with cerebral tau burden. It is possible that changes in blood do not always reflect changes in the brain, so follow-up research is needed that specifically examines the implications of this work to cerebral tauopathy.

### Clinical implications

Since elevated pTau-181 levels often predict cognitive decline, this study might imply that the long-term prognosis for participants with N-PASC may be poorer in patients with increased pTau-181. Long-term studies are required to determine whether pTau-181 levels will continue to rise or will stabilise over time in participants with N-PASC. Additionally, prognostic studies are necessary that determine if pTau-181 increases are predictive of subsequent cognitive decline and impairment in N-PASC. However, the finding that changes in pTau-181 are independent of changes in NfL and GFAP might support the view that N-PASC is a heterogeneous condition with different symptoms that might indicate independent neuropathological processes. Thus, if cerebral tau is present, then results could highlight the potential for neuroprotective strategies including anti-inflammatory or anti-Tau therapies to help mitigate COVID-19-related cognitive decline.^53^ Future research is warranted that elucidates the mechanisms through which COVID-19 influences Tau phosphorylation and assesses the long-term prognostic implications of pTau-181 elevation in at-risk populations.

## Contributors

Clouston, Yang, and Luft had full access to all the data in the study and take responsibility for the integrity of the data and accuracy of the data analysis. Study concept and design: Yang, Clouston, Luft. Acquisition, analysis, and interpretation of data: Yang, Clouston, Fontana. Drafting of the manuscript: Clouston, Yang. Critical revision of the manuscript for important intellectual content: All Authors. Statistical analysis: Clouston. Obtained funding: Clouston, Luft. Administrative, technical, or material support: Fontana, Yang. Study supervision: Luft. All authors read and approved of the final version of the manuscript.

## Data sharing statement

These data represent private health information that also include potentially identifiable longitudinal data. After publication, a limited dataset can be shared with bona fide researchers upon receipt of a written request by email and after executing a data use agreement. Analytic code relies on standard packages available from Stata MP V.17, and detailed analyses will be shared with researchers via the open science framework (osf.io).

## Declaration of interests

The authors have no conflicts of interest to disclose.
