# Supplementary material for: Increased phosphorylated tau (pTau-181) is associated with neurological post-acute sequelae of coronavirus disease in essential workers: a prospective cohort study before and after COVID-19 onset
Source: eBioMedicine. 2026 Jan 5;123:106106. doi: 10.1016/j.ebiom.2025.106106 (PMC12811499; doi:10.1016/j.ebiom.2025.106106)
Supplement: Supplemental Materials [file mmc1.docx]

**Supplemental Appendix**

**Supplemental Figure 1.** Histogram showing the temporal distribution of cases included in the present study

**Supplemental Figure 2.** Flowchart depicting the selection of study participants

**Supplemental Table 1**. Full results for ln-Gamma models of phosphorylated tau-181 examining change in pTau-181 levels over time compared to pre-COVID-19 levels, adjusting for within-individual random intercepts

**Supplemental Table 2**. Overlapping cell percent of sample that exhibited ≥change in target dysregulated biomarkers among participants with neurological post-acute sequelae of coronavirus disease 2019 (N-PASC), by type

**Supplemental Figure 1.** Histogram showing the temporal distribution of cases included in the present study

**Supplemental Figure 2.** Flowchart depicting the selection of study participants

**Supplemental Table 1**. Full results for ln-Gamma models of phosphorylated tau-181 examining change in pTau-181 levels over time compared to pre-COVID-19 levels, adjusting for within-individual random intercepts

| Variable | Coef. | SE | P |
| --- | --- | --- | --- |
| Pre-COVID-19 | Reference | | |
| Controls at follow-up | 0.12 | 0.03 | <0.001 |
| Onset of N-PASC | 0.24 | 0.03 | <0.001 |
| Age, years | 0.01 | 0.00 | <0.001 |
| Female Sex/Gender | -0.19 | 0.09 | 0.028 |
| Blood Volume | -0.01 | 0.00 | 0.054 |
| Intercept | 0.76 | 0.25 | 0.002 |
| Log(SD) | -1.13 | 0.03 | <0.001 |
| Random Intercept (SD) | 0.15 | 0.01 | <0.001 |
| Akaike Information Criterion |  | 1787.1 |  |
| Bayesian Information Criterion |  | 1820.7 |  |
| Likelihood Ratio Test comparing to linearity |  | 236.3 | <0.001 |

Abbreviations: COVID-19: coronavirus disease 2019; N-PASC: neurological post-acute sequelae of COVID-19; SD: standard deviation; SE: standard error; Coef.: regression coefficient.

**Supplemental Table 2**. Overlapping cell percent of sample that exhibited ≥change in target dysregulated biomarkers among participants with neurological post-acute sequelae of coronavirus disease 2019 (N-PASC), by type

| Decrease Variables | No Increase in pTau-181 | ≥20% Increase in pTau-181 | Total |
| --- | --- | --- | --- |
| No Decrease ≥20% in GFAP or NfL | 24.6 | 36.2 | 60.7 |
| Decrease ≥20% in GFAP | 6.7 | 7.1 | 13.8 |
| Decrease ≥20% in NfL | 5.8 | 8.9 | 14.7 |
| Decrease ≥20% in both GFAP and NfL | 5.4 | 5.4 | 10.7 |
| Total | 42.4 | 57.6 | 100.0 |

Abbreviations: GFAP: glial fibrillary acidic protein; NfL: neurofilament-light; pTau-181: phosphorylated tau 181.
